# Immune dysregulation syndrome associated with inborn errors of metabolism – hemophagocytic lymphohistiocytosis in the context of isovaleric acidemia: a case report

**DOI:** 10.3389/fped.2026.1805260

**Published:** 2026-06-12

**Authors:** Frances Fuenmayor, Santiago Chávez, Sofía Ortíz, Marcelo Guerrero, Melanie Orellana, Diego Veintimilla, Inés Fernández, Leonel Meza

**Affiliations:** 1 Baca Ortiz Pediatric Hospital, Quito, Ecuador; 2 School of Medicine, UDLA School of Medicine, Quito, Ecuador; 3 Intensive Care Medicine department, Pontifical Catholic University, Quito, Ecuador

**Keywords:** anti-N-Methyl-D-aspartate receptor encephalitis, hemophagocytic lymphohistiocytosis, immune system diseases, inborn errors, isovaleric acidemia, metabolism

## Abstract

Hemophagocytic lymphohistiocytosis (HLH) continues to pose a diagnostic challenge in pediatric critical care, not only because of its severity but also due to the wide range of conditions that can underlie its presentation. Among these, metabolic disorders are often overlooked. We describe an 18-month-old boy who initially presented with persistent fever and respiratory symptoms, later evolving to pancytopenia, hepatosplenomegaly, and neurological deterioration, the patient fulfilled seven of the eight HLH-2004 criteria. Along the way, adenovirus and parainfluenza virus type III were identified, and anti-NMDA receptor encephalitis was confirmed, adding further complexity to the clinical picture. What proved decisive was the identification of a homozygous pathogenic variant in the IVD gene (c.1174C > T; p.Arg392Cys), establishing the diagnosis of isovaleric acidemia. This finding reframed the case. Rather than an isolated hyperinflammatory syndrome, the clinical course can be better understood as the result of a metabolic disorder capable of amplifying immune dysregulation, particularly in the setting of intercurrent infection. In this context, the features of HLH appear not as a separate entity but as part of a broader process, where metabolic decompensation, accumulation of toxic intermediates, and systemic inflammation converge. This overlap is likely underrecognized in clinical practice. Recognizing this possibility has practical implications. It shifts the diagnostic focus, but also opens the door to more tailored management strategies. In similar cases, HLH may be less an endpoint diagnosis and more a signal pointing toward an underlying metabolic or genetic condition that requires specific attention.

## 1 Introduction

Hemophagocytic lymphohistiocytosis (HLH) is a life-threatening hyperinflammatory syndrome driven by uncontrolled macrophage and cytotoxic T-lymphocyte activation, culminating in multiorgan failure. Diagnosis rests on the HLH-2004 protocol (≥ 5 of 8 criteria): fever, splenomegaly, cytopenias in ≥ 2 lineages, hypertriglyceridemia or hypofibrinogenemia, hemophagocytosis on bone marrow biopsy, reduced NK-cell activity, hyperferritinemia (> 500 μg/L), and elevated soluble CD25. HLH is classified as primary (genetic, due to biallelic mutations in cytotoxic pathway genes such as PRF1, UNC13D, or RAB27A) or secondary (triggered by viral infections, malignancies, or autoimmune disease). Secondary HLH predominates in pediatric populations. Inborn errors of metabolism (IEM) represent an underrecognized trigger, where catabolic stress and systemic inflammation during metabolic crises may drive secondary hyperinflammatory responses.

This report describes a pediatric patient fulfilling HLH-2004 criteria in the context of concurrent adenovirus and parainfluenza type III co-infection, anti-NMDAR autoimmune encephalitis, and a homozygous pathogenic IVD variant confirming isovaleric acidemia. We examine the role of this metabolic defect as a trigger for secondary HLH and discuss the therapeutic implications.

### 1.1 Clinical case presentation

An 18-month-old boy presented to a private clinic in June 2025 with a five-day history of persistent fever. Initially managed as unspecified tonsillitis, he showed no response to parenteral antibiotics and was subsequently transferred to a local hospital, where pneumonia of undetermined etiology and encephalopathy—possibly related to the respiratory infection—were diagnosed.

Four days later, he was referred to the pediatric trauma and shock unit due to progressive respiratory and neurological deterioration accompanied by significant gastrointestinal symptoms. On admission, he scored 9/15 on the Glasgow Coma Scale, exhibited generalized hypotonia, and showed no meningeal signs. Vital signs remained relatively stable with supplemental oxygen requirement.

Given worsening respiratory compromise, invasive mechanical ventilation and broad-spectrum empirical antimicrobial coverage were initiated. PCR-based microbiological studies of sputum samples were positive for Adenovirus and Parainfluenza virus type III, while CSF analysis was negative. Significant elevations in procalcitonin and interleukin-6 were documented, indicative of systemic inflammatory response.

Magnetic resonance neuroimaging revealed abnormalities in cortico-subcortical differentiation and lesions consistent with encephalitis. Subsequently, complete blood count showed persistent pancytopenia and elevated ferritin levels, associated with hepatosplenomegaly. Bone marrow aspiration confirmed hemophagocytosis, establishing the diagnosis of HLH.

A history of a sibling who died from a similar clinical presentation prompted investigation for an underlying genetic or metabolic disease. The patient received comprehensive intensive care management for 11 days, with favorable outcomes after intensive support and immunomodulation, followed by ongoing multidisciplinary follow-up.

## 2 Methodology

A multimodal diagnostic workup was performed, comprising: (i) serological and immunological studies (immunoglobulin quantification, anti-NMDAR antibodies in CSF, bone marrow flow cytometry); (ii) microbiological studies [lower respiratory tract multiplex PCR (GeneXpert), CSF culture and PCR]; (iii) cytological/histopathological examination of bone marrow aspirate for hemophagocytosis; (iv) biochemical panel (complete blood count, hepatobiliary enzymes, ferritin, fibrinogen, IL-6, procalcitonin); and (v) multisequence brain MRI (DWI, T1, T2-FLAIR, contrast-enhanced).

Genetic analysis was performed by whole-exome sequencing (WES) using next-generation sequencing (NGS) at Hospital de Especialidades Eugenio Espejo, Quito, Ecuador (September 2025). The panel included comprehensive analysis of genes associated with Inborn Errors of Immunity (IEI), including STAT3, CTLA4, and LRBA, as well as genes associated with inborn errors of metabolism.

Study limitation: Due to local diagnostic constraints, biochemical confirmation via plasma acylcarnitine profile or urinary organic acid analysis was not performed. However, the identification of a known pathogenic variant in the IVD gene, coupled with the patient's sepsis-like presentation, cytopenias, and family history, strongly supports a metabolic crisis as the primary driver of the hyperinflammatory state. The clinical phenotype is consistent with reported cases where organic acidemias act as triggers for secondary HLH.

## 3 Results

Laboratory findings and diagnostic assessments are summarized extensively in Table 1 and Tables 2–6. Briefly, the complete blood count confirmed pancytopenia (Hb 8.5 g/dL, platelets 10,000/μL, leukocytes 0.8 × 10^3^/mm^3^ with severe lymphopenia 0.2 × 10^3^/mm^3^). Ferritin was markedly elevated at 755 μg/L, fulfilling the HLH-2004 threshold, with IL-6 also elevated at 97.40 pg/mL. Immunoglobulin levels were low normal: IgG 484 mg/dL (reference 345–1,213 mg/dL for age 12–24 months) and IgA 22.4 mg/dL (reference 14–106 mg/dL for age 12–24 months). It should be noted that IgA values are generally considered reliable only after 4 years of age. This pattern likely reflects an inadequate, transient humoral response or capillary leak in the context of acute severe HLH, rather than a primary humoral immunodeficiency, an interpretation supported by the patient's clinical improvement following IVIG replacement.

**Table 1:** HLH-2004 diagnostic criteria assessment.

Criterion	Finding in this Patient	Threshold	Met?
1. Fever	Persistent fever >38.5 °C	≥38.5 °C	Yes
2. Splenomegaly	Hepatosplenomegaly on imaging	Present	Yes
3. Cytopenias (≥2 lineages)	Hb 8.5 g/dL; Plt 10,000/μL; Neutrophils 0.8 × 10^3^/mm^3^	Hb <9/Plt <100 K/ANC <1K	Yes
4. Hypertriglyceridemia or Hypofibrinogenemia	Fibrinogen 155.9 mg/dL (borderline low)	Fibrinogen <150 mg/dL or TG ≥3.0 mmol/L	Borderline
5. Hemophagocytosis in bone marrow	Confirmed on bone marrow aspirate	Present	Yes
6. Hyperferritinemia	Ferritin 755 μg/L	>500 μg/L	Yes
7. Low/absent NK-cell activity	Not available locally	Decreased or absent	Not tested
8. Elevated soluble CD25 (sIL-2R)	IL-6 markedly elevated (97.4 pg/mL); sCD25 not measured	>2,400 U/mL	Surrogate elevated

**Table 2:** Analytical findings and data interpretation.

Parameter	Finding	Interpretation
Hemoglobin	8.5 g/dL	Anemia – meets HLH criterion
Platelets	10,000/μL	Severe thrombocytopenia – meets HLH criterion
Leukocytes/Lymphocytes	0.8 × 10^3^/mm^3^; Lymphocytes 0.2 × 10^3^/mm^3^	Leukopenia + severe lymphopenia
Ferritin	755 μg/L	Elevated (> 500 μg/L) – meets HLH criterion
Fibrinogen	155.9 mg/dL	Approaching borderline low
IL-6	97.40 pg/mL	Marked systemic inflammatory response
Anti-NMDAR antibodies (CSF)	3.1 IU/mL	Positive – confirms autoimmune encephalitis
IgG/IgA	IgG 484 mg/dL (ref. 345–1,213 mg/dL)/IgA 22.4 mg/dL (ref. 14–106 mg/dL)^a^	Low-normal levels that were inappropriately low for the degree of inflammation
PCR lower respiratory tract	Parainfluenza III + Adenovirus	Viral co-infection – HLH trigger
Procalcitonin	4.57 ng/mL	Severe systemic inflammatory response
AST/ALT/Amylase	157/135/870 U/L	Hepatic and pancreatic involvement
IVD gene (WES)	c.1,174C > T (p.Arg392Cys) – homozygous	Pathogenic – confirms Isovaleric Acidemia
STAT3, CTLA4, LRBA (WES)	No pathogenic variants identified	Primary immunodeficiency excluded

^a^ Reference range for age 12–24 months; note that serum IgA values are generally considered clinically reliable only after 4 years of age.

**Table 3:** Flow cytometry immunophenotyping.

Test	Findings	Interpretation
Flow cytometry (bone marrow)	Erythroid series present; Neutrophils 66.6%; Monocytes 9.9%; T lymphocytes 16.6%; NK cells 0.32%; B lymphocytes 2.1%	No phenotypic alterations for neoplastic markers studied. No evidence of pathological cell infiltration.

**Table 4:** Cytological findings of bone marrow aspirate.

Test	Finding	Interpretation
Bone marrow aspirate	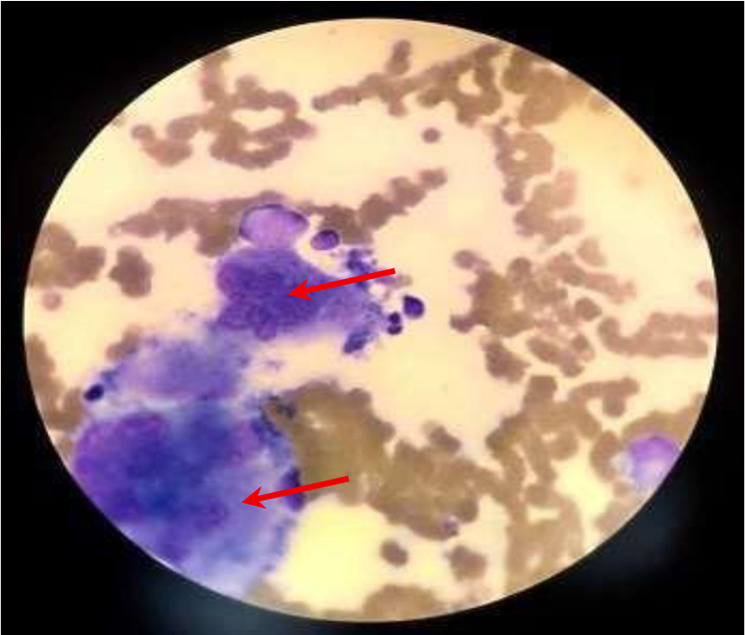 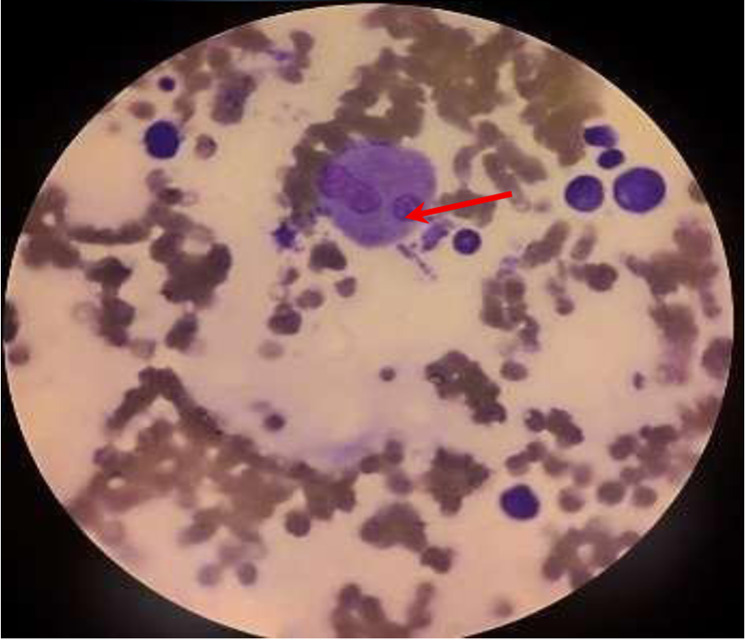 Hemophagocytosis: macrophages engulfing leukocytes, erythrocytes, and platelets	Confirms secondary hemophagocytic syndrome – meets HLH-2004 criterion #5

**Table 5:** Supportive and pharmacological interventions applied.

Drug/Intervention	Administration	Duration	Rationale	Outcome
Ceftriaxone	IV	4 days (Jun 19–23)	Empirical sepsis/gram-negative coverage	Discontinued on improved infectious profile
Vancomycin	IV	11 days (Jun 19–30)	Resistant gram-positive/suspected meningitis	Maintained during prolonged febrile course
Meropenem	IV	7 days (Jun 23–30)	Suspected nosocomial/resistant infection; elevated PCT; pancytopenia	Completed course
TMP-SMX	IV	7 days (Jun 23–30)	Prophylaxis for immunodeficiency under investigation/HLH	Maintained
Acyclovir	IV	2 days (Jun 23–25)	Suspected viral meningoencephalitis	Discontinued after negative viral studies
Oseltamivir	Oral	2 days (Jun 23–25)	Empirical viral respiratory	Discontinued: influenza ruled out
Fluconazole	IV	5 days (Jun 25–30)	Fungal prophylaxis (immunosuppression)	Maintained
Immunoglobulin IVIG	IV	Unspecified	Immune modulation/ supportive therapy	Clinical improvement
IMV with ETT	ETT 3.5 mm FiO2 30%–65%	7 days (Jun 19–26)	Acute respiratory failure	Successful weaning on Day 7
NIV	Mask/Cannula	4 days (Jun 26–30)	Post-extubation transition	Successful
Midazolam + Fentanyl	IV continuous infusion	Duration of IMV	Deep sedation/analgesia	Gradual withdrawal on recovery
Dexmedetomidine	IV 0.3–1 μg/kg/h	Post-extubation	Light sedation/neuroprotection	Adjusted per consciousness level

**Table 6:** Brain MRI findings by sequence.

Sequence	Finding	Interpretation
DWI	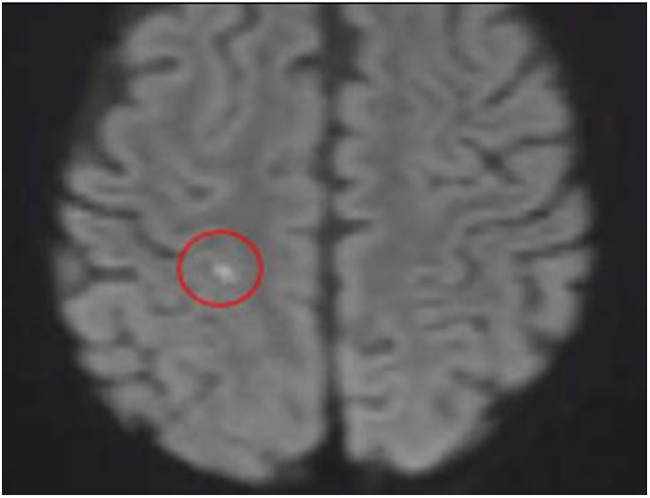 Minimal right subcortical postcentral parietal hyperintensity; restricted diffusion and signal drop on ADC map	Hyperacute ischemic focus
T2-FLAIR (axial)	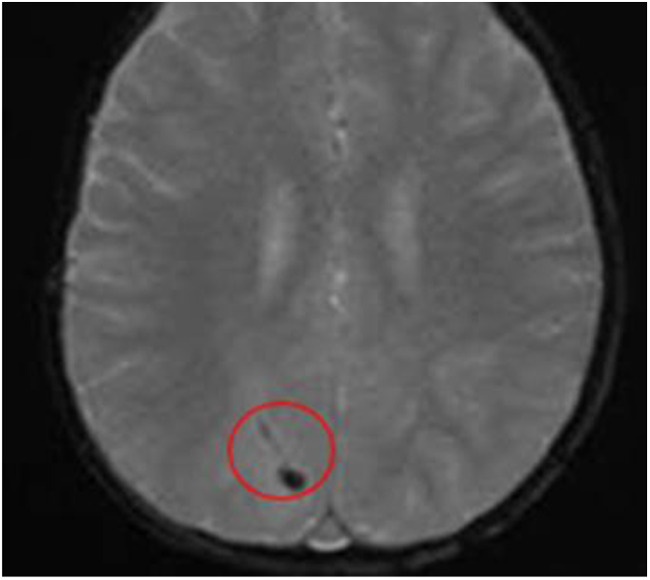 Hypointense spots in right occipital region; hyperintense subcortical white matter in bilateral occipital and parieto-occipital regions	Hemorrhagic collection; encephalitis pattern
T1/ADC (contrast-enhanced)	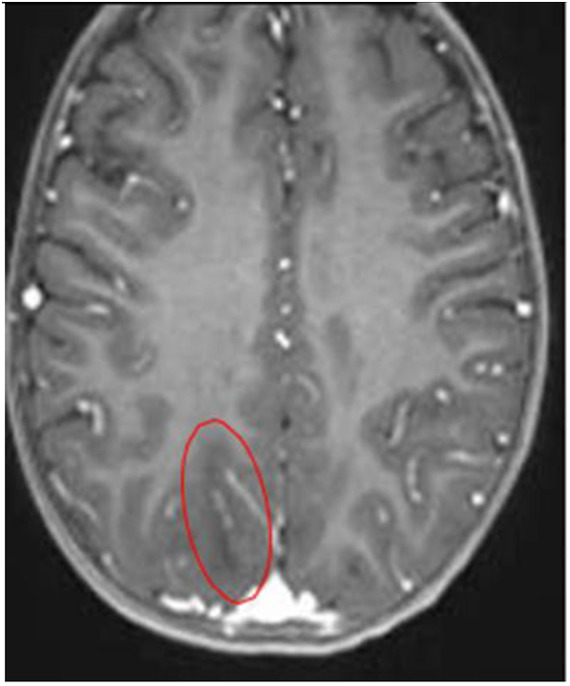 Hypointense focus in right subcortical postcentral parietal region; probable demyelination areas	Demyelination/PRES pattern
T2-FLAIR (posterior fossa)	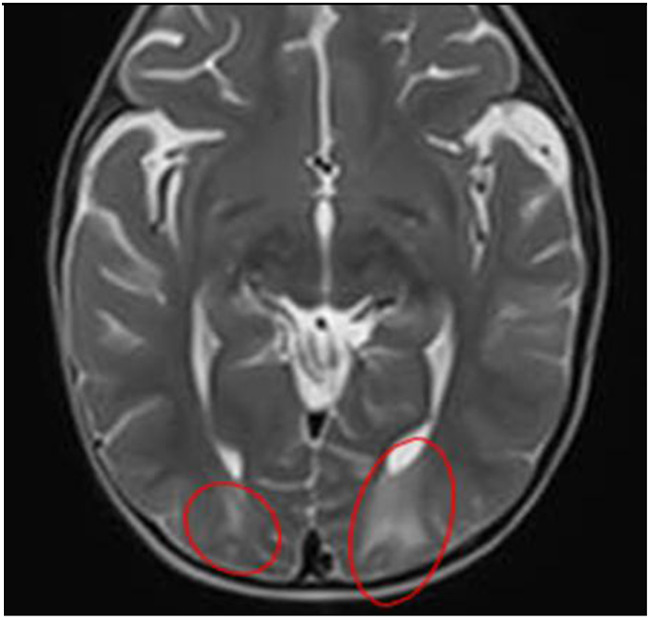 Abnormalities in gray-white matter differentiation; bilateral maxillary sinus hyperintensity	Encephalitis.

Microbiological and immunological evaluation confirmed a Parainfluenza virus type III and Adenovirus lower respiratory co-infection. While the cerebrospinal fluid was negative for direct pathogens, anti-NMDAR antibodies were detected at a titer of 3.1 IU/mL, establishing the diagnosis of anti-NMDA receptor autoimmune encephalitis. Cytological examination of the bone marrow aspirate confirmed hemophagocytosis, while flow cytometry ruled out neoplastic infiltration. Brain MRI demonstrated hyperacute ischemic changes, abnormalities in gray-white matter differentiation consistent with encephalitis, and probable demyelinating foci.

Genetic analysis via whole-exome sequencing identified a pathogenic homozygous IVD variant [c.1174C > T; p.Arg392Cys (NM_002225.5); ClinVar ID: 265202], confirming the diagnosis of isovaleric acidemia. The panel explicitly excluded primary immunodeficiencies, with no pathogenic variants identified in IEI-associated genes, including STAT3, CTLA4, and LRBA. Ultimately, the patient fulfilled 7 of the 8 HLH-2004 diagnostic criteria.

## 4 Discussion

In general terms, the presence of hemophagocytic syndrome (HPS/HLH) is typically associated with a poor prognosis. Secondary forms of HPS, commonly linked to infections (usually viral), neoplasms, or autoimmune processes, are more frequent than primary forms. In our patient, seven of eight HLH-2004 criteria were met, with histologically confirmed hemophagocytosis. The Adenovirus and Parainfluenza III co-infection likely served as the acute inflammatory trigger, consistent with the pediatric cohort reported by Astudillo et al., in which similar viral co-infections were identified using broad microbiological panels.

This case illustrates the pathophysiological interplay between isovaleric acidemia and secondary HLH. The homozygous IVD variant provides a unifying explanation for the clinical phenotype — recurrent infections, thrombocytopenia, and a family history of early death with a similar presentation — pointing to autosomal recessive inheritance. The relevance of this case lies in the clinical and pathophysiological overlap between the metabolic crises of isovaleric acidemia and secondary HLH. During metabolic decompensations, patients with isovaleric acidemia experience intense catabolic stress, systemic inflammation, mitochondrial dysfunction, and accumulation of toxic metabolites (particularly isovaleryl-CoA and isovaleric acid), which may manifest as cytopenias, hepatic impairment, multiorgan failure, and a sepsis-like presentation. This scenario can mimic an HLH-like phenotype or, in extreme cases, act as a trigger for secondary HLH, particularly with concurrent infections. The described thrombocytopenia constitutes a key finding and a point of convergence between severe metabolic crises and HLH criteria.

From an epidemiological standpoint, isovaleric acidemia is a rare disease, with geographic variability. When organic acidemias are considered as a group, their incidence increases; population-based neonatal screening studies have reported rates close to 1/10,000 newborns, underscoring their clinical relevance in pediatrics.

Beyond the classical primary and secondary HLH categories, a growing body of evidence highlights that inborn errors of immunity (IEI) other than familial HLH, and inborn errors of metabolism (IEM), may present with or trigger HLH, often preceding a definitive diagnosis of the underlying disease. A systematic review of 178 pediatric patients identified 46 distinct IEI associated with HLH, with combined immunodeficiencies and immune dysregulation disorders most frequently implicated; notably, HLH preceded the IEI diagnosis in 75% of cases, underscoring its value as an early warning sign (23). Among specific monogenic disorders, deficiency of adenosine deaminase 2 (DADA2) can manifest primarily as recurrent viral infections and secondary HLH — as illustrated by a case in which these features were the initial and dominant presentations before the underlying diagnosis was established (24). In parallel, IEM such as glutaric aciduria type IIC have been reported to trigger HLH-like features, with evidence suggesting that targeted treatment of the primary metabolic disorder may be pivotal in resolving the hematological syndrome (1–25). Taken together, approximately 10% of HLH cases may be attributable to rare underlying diseases, including IEI and IEM, emphasizing the need for broad diagnostic evaluation — encompassing metabolic and genetic studies — in any pediatric patient presenting with HLH, particularly when conventional triggers are absent or the clinical course is atypical (26).

Primary immunodeficiency was molecularly excluded by WES, confirming the IVD metabolic defect as the sole genetic driver. The family history of a sibling's death with a similar presentation is most parsimoniously explained by shared homozygosity for the IVD variant under autosomal recessive inheritance. The detection of anti-NMDAR antibodies in CSF confirmed the coexistence of anti-NMDA receptor autoimmune encephalitis. This entity originates etiopathogenically from antibodies against the GluN1 subunit of the NMDAR receptor, causing profound synaptic dysfunction and glutamatergic system hypofunction. In the systematic review by Yam et al., which included 283 pediatric patients, the most common presentations were seizures and extrapyramidal symptoms, with both EEG and MRI showing abnormalities in a significant proportion; these findings align with those observed in our case. The respiratory viral prodrome likely triggered molecular mimicry or post-infectious autoimmune activation leading to NMDAR antibody production.

The presence of posterior reversible encephalopathy syndrome (PRES) should also be considered. In our patient, neurological hypoactivity correlated with cerebral edema on MRI justifies this diagnosis; this was supported by the presence of hematologic disease and immunosuppressive drugs as risk factors. Early recognition of PRES is key, as its course can be reversed if the underlying cause is addressed.

General and primary treatment measures included progressive antimicrobial coverage, neuroprotective sedation, early ventilatory support, and prompt enteral nutrition. Replacement therapy with immunoglobulins was employed with clinical improvement. Future therapeutic choices must be based on the specific genotype and the altered metabolic/immunological pathway. The patient's clinical improvement following 11 days of intensive support highlights the efficacy of early, multidisciplinary, and metabolically guided management. However, the risk of recurrence during future metabolic decompensations remains high; thus, close metabolic and neurological follow-up is required, and dietary management with leucine restriction and emergency regimens must be maintained.

This case report has important limitations. Biochemical confirmation of isovaleric acidemia could not be established during the acute admission due to the lack of local availability of plasma acylcarnitine profiling by tandem mass spectrometry and urinary organic acid analysis. Therefore, the diagnosis of isovaleric acidemia was established molecularly through whole-exome sequencing, but the acute metabolic state could not be biochemically characterized. Additionally, certain specialized tests for hemophagocytic lymphohistiocytosis (HLH), such as soluble CD25 levels and natural killer cell activity, were not available. Measurement of triglyceride levels and serial fibrinogen should be incorporated when feasible. Adenovirus was identified in the lower respiratory tract; however, the absence of blood adenoviral load assessment limits the evaluation of systemic viral involvement. Finally, the neurological findings likely reflect overlapping pathophysiological mechanisms, including autoimmune encephalitis, posterior reversible encephalopathy syndrome (PRES), HLH-associated central nervous system inflammation, ischemia, and metabolic encephalopathy. Also, the prevalence of HLH among patients with isovaleric acidemia is unknown, and the association remains poorly established. This case should therefore be interpreted as a rare potential overlap rather than evidence of a recognized frequent complication.

This case highlights the importance of considering inborn errors of metabolism in pediatric patients presenting with HLH-like syndromes, particularly when systemic inflammation is accompanied by encephalopathy, pancytopenia, hepatosplenomegaly, gastrointestinal symptoms, and a suggestive family history. Although a direct causal relationship between isovaleric acidemia and HLH cannot be definitively established in this case, their coexistence suggests a clinically meaningful overlap. Recognizing it requires a hierarchical diagnostic approach in which an underlying metabolic disorder is actively sought when the clinical course exceeds that expected for isolated metabolic decompensation — a recognition that has direct implications for therapeutic decision-making, prognostic stratification, and potential mortality reduction. Future reports should incorporate comprehensive metabolic profiling, serial HLH biomarkers, bone marrow evaluation, viral load assessment, and detailed treatment response to further characterize this rare but clinically significant association.

## Data Availability

The original contributions presented in the study are included in the article and its Supplementary Material. The specific genetic variant identified in this case report is publicly accessible in the ClinVar database under the accession ID 265202. Due to ethical and privacy restrictions concerning identifiable human clinical and genomic data, the raw whole-exome sequencing (WES) datasets are not publicly available. Requests to access the restricted datasets should be directed to the corresponding author, Frances Fuenmayor.
