# Supplementary material for: Immune dysregulation syndrome associated with inborn errors of metabolism – hemophagocytic lymphohistiocytosis in the context of isovaleric acidemia: a case report
Source: Front Pediatr. 2026 Jun 12;14:1805260. doi: 10.3389/fped.2026.1805260 (PMC13303336; doi:10.3389/fped.2026.1805260)
Supplement: Supplementary file 1 [file Datasheet1.pdf]

EXOMA COMPLETO NGS

HBO25-077

Análisis de Exoma Completo por NGS

INFORME PRELIMINAR

HOSPITAL DE ESPECIALIDADES EUGENIO ESPEJO

Profesional Solicitante: --------------------------------

DATOS DEL PACIENTE

Nombres: ——————————————————-

Número único de identidad: ----------------------------

Edad: 1 años

Sexo: Masculino

Tipo de Muestra: Sangre periférica

Fecha de recepción de la muestra: 26/06/2025

Fecha del reporte: 12/09/2025

1. MOTIVO DE LA SOLICITUD

Infecciones recurrentes + trombocitopenia + hermano fallecido con cuadro similar.

2. RESULTADOS

POSICIÓN	CAMBIO DE NUCLEÓTIDO	CAMBIO EN LA PROTEÍNA	EFECTO	GENOTIPO	GENOMA DE REFERENCIA	RS	CLASIFICACIÓN ACMG
IVD NM_002225.5							
chr15:40418165 C > T	c.1174C>T	p.(Arg392Cys)	Missense	Hom	GRCh38	rs4924466	5
MAPKBP1 NM_014994.3							
chr15:41813750 G > A	c.949G>A	p.(Gly317Arg)	Missense	Hom	GRCh38	rs2064844218	3

5 Patogénica   4 Probablemente Patogénica   3 Significado incierto (VUS)

Detalles de la variante

1. La variante c.1174C>T, en homocigosis, en el gen *IVD* (OMIM 607036), que promueve la sustitución de citosina por timina, provocando el reemplazo de arginina por cisteína en la proteína p.(Arg392Cys). Esta variante está presente en bases de datos poblacionales (rs371427844, gnomAD 0,007%). Este cambio de sentido se ha observado en individuos con acidemia isovalérica (PMID: 9665741, 17027310, 25220015, 26018748). Esta variante también se conoce como 1174 C>T (Arg363Cys). ClinVar contiene una entrada para esta variante (Variation ID: 265202). Un algoritmo desarrollado para predecir el efecto en la estructura y función de la proteína (PolyPhen-2) sugiere que es probable que esta variante sea disruptiva. Estudios experimentales han demostrado que esta variante missense afecta la función del IVD (PMID: 9665741). Se ha determinado que otras variantes que alteran este residuo son patogénicas (PMID: 22960500, 25220015, 27904153). Por estas razones, esta variante se ha clasificado como patogénica.

2. La variante c.949G>A, en homocigosis, en el gen *MAPKBP1* (OMIM 616786), que promueve la sustitución de guanina por adenina, provocando el reemplazo de glicina por arginina en la proteína p.(Gly317Arg). Se trata de una variante extremadamente rara en bancos de datos poblacionales. Esta variante no se ha descrito en individuos afectados con condiciones asociadas a *MAPKBP1.* De acuerdo a las recomendaciones del American College of Medical Genetics (ACMG), la variante se clasifica como de significado clínico incierto (VUS).

Interpretación Biológica y Clínica

1. Las variantes patogénicas en homocigosis o en heterocigosis compuesta en el gen IVD se asocian a Acidemia Isovalérica (IVA, OMIM 243500), un error innato del metabolismo de la leucina causado por una deficiencia de la enzima isovaleril-CoA deshidrogenasa. Puede presentarse con cetoacidosis neonatal grave que puede causar la muerte, pero en casos más leves se presentan episodios recurrentes de cetoacidosis de diversa gravedad posteriormente, en la infancia y la niñez.

   La IVA clásica se caracteriza por descompensaciones metabólicas agudas (vómitos, mala alimentación, letargia, hipotonía, convulsiones y un olor característico a pies sudorosos). Estas descompensaciones metabólicas agudas suelen desencadenarse por ayuno, enfermedades (febriles) (especialmente gastroenteritis) o un aumento de la ingesta de proteínas. El deterioro clínico suele ocurrir en cuestión de horas o días después del nacimiento. Otras manifestaciones de la IVA clásica incluyen retraso en el desarrollo, discapacidad intelectual o deterioro cognitivo, epilepsia y trastornos del movimiento (temblor, dismetría, movimientos extrapiramidales). Se trata de una condición de herencia autosómica recesiva. Se sugiere la realización de asesoramiento genético.

2. Las variantes patogénicas en homocigosis o en heterocigosis compuesta en el gen *MAPKBP1* causan Nefronoptisis 20 (NPHP, OMIM 617271), autosómica recesiva. Se trata de una nefritis tubulointersticial que se caracteriza por fibrosis renal progresiva que resulta en insuficiencia renal terminal. La edad de inicio es relativamente tardía en comparación con otras formas de NPHP, y los pacientes desarrollan enfermedad renal terminal en la segunda o tercera décadas de la vida. A diferencia de la mayoría de las otras formas de NPHP, la NPHP20 no presenta características de ciliopatía y los pacientes no parecen presentar manifestaciones extrarrenales. Como la variante identificada es de significado clínico incierto, no es posible confirmar ni descartar el diagnóstico de Nefronoptisis 20. Se sugiere la realización de asesoramiento genético.

Se resalta que el análisis de variaciones en el número de copias (CNVs) por secuenciación de nueva generación tiene sensibilidad y especificidad reducidas.

Panel de genes priorizados para el análisis:

ISCA2, PGAP1, PGAP2, PGAP3, GORAB, XYLT2, XYLT1, NFU1, C1GALT1C1, ATP6V1E1, GLUL, SEC63, ALG8, COG8, ALG9, COG7, ALG6, COG6, COG5, COG4, SLC35C1, ALG2, COG2, ALG3, COG1, MOGS, ALG1, CYP27A1, BOLA3, GNE, LIPT2, LIPT1, (TPP1), RPN2, SLC35D1, SAR1B, B3GLCT, LIAS, GNS, ATP6V0A2, ST3GAL5, B4GALNT1, CHST14, ST3GAL3, B3GALNT2, NUS1, SLC10A7, TGDS, PMM2, EXT1, TMEM199, EXT2, B4GAT1, SLC6A9, PPT1, ALDH18A1, PIGU, PIGT, PIGO, GPAA1, PIGN, HEXB, PIGQ, HEXA, PIGP, MAGT1, PIGW, PIGV, PIGY, LFNG, NANS, CTSD, CHST6, SSR4, CCDC115, ACAD9, SSR3, PIGC, PIGB, SLC7A7, PIGA, IVD, PIGM, PIGL, ISPD, PIGG, CHST3, IBA57, TRAK1, PAPSS2, TMEM165, MGAT2, EXTL3, SLC16A1, ARCN1, VMA21, TRIP11, DPAGT1, POGLUT1, TUSC3, MPI, GNPTAB, NAGLU, POFUT1, TRAPPC12, RXYLT1, TRAPPC11, SLC39A8, LARGE1, CSGALNACT1, ALG14, ALG13, ALG12, ALG11, DHDDS, KCTD7, DPM1, DPM2, DPM3, SLC9A7, EOGT, ATP6V1A, COPB2, COPA, SLC26A2, FUT8, FUK, MPDU1, TRAPPC2, GFPT1, SRD5A3, B3GALT6, DOLK, TRAPPC9, G6PC3, STT3A, DHCR7, STT3B, OGT, POMT2, B4GALT1, PRKCSH, NGLY1, POMT1, SGSH, GMPPB, MFSD8, GM2A, CA5A, DSE, GMPPA, PGM3, HMGCS2, A4GALT, SLC37A4, HGSNAT, JAGN1, ATP6AP1, GLRX5, ATP6AP2, CAD, VPS13B, POMGNT2, POMGNT1, DDOST, NPC1, NPC2, TRAPPC6B, RFT1, MAN1B1, B4GALT7, SEC23A, CANT1, GOSR2, CLN8, CLN6, CLN5, CLN3, CLN2, GANAB, CHSY1, OXCT1, D2HGDH, SEC23B, FKTN, SLC35A2, SLC35A1, FKRP, SLC35A3, GALNT3, B3GAT3, POMK, ATP13A2, SEC24D

3. HALLAZGOS SECUNDARIOS

Como un hallazgo secundario, se identificó, en heterocigosis en el gen TTN (OMIM 188840) la variante c.36119-1G>C, localizada en región de splicing. Los programas informáticos de predicción ´´in silico´´ sobre el splicing sugieren que interrumpa el splicing del ARN y probablemente resulte en una proteína TTN truncada. Esta variante no está presente en bases de datos poblacionales (gnomAD sin frecuencia). ClinVar contiene una entrada para esta variante (Variation ID: 1321432).

Esta variante se encuentra en la banda I de TTN (PMID: 25589632). Se han reportado variantes truncadas en esta región en individuos afectados con miopatía centronuclear autosómica recesiva (PMID: 23975875). También se han identificado variantes truncadas en esta región en personas con miocardiopatía dilatada autosómica dominante o condiciones cardiovasculares (PMID: 27869827, 32964742). La evidencia por el momento es insuficiente, sin embargo, se clasifica esta variante como probablemente patogénica para condiciones recesivas.

Lista ACMG SF v3.2 de hallazgos secundarios[4]:

RET, RB1, ACVRL1, RYR1, RYR2, HFE, PTEN, PCSK9, PRKAG2, BRCA1, BRCA2, STK11, ATP7B, TMEM43, SCN5A, MEN1, DSP, MYBPC3, KCNH2, TNNC1, GAA, TPM1, TSC2, SDHC, TSC1, SDHD, HNF1A, SDHB, TGFBR1, TGFBR2, TRDN, MSH6, ACTA2, BTD, SDHAF2, MSH2, TNNT2, MYL2, KCNQ1, MYL3, PKP2, CASQ2, RBM20, DSG2, TP53, DSC2, MYH7, ENG, MAX, TTN, TTR, TMEM127, BAG3, LMNA, PMS2, MYH11, TNNI3, VHL, FLNC, CACNA1S, APOB, LDLR, MUTYH, SMAD4, SMAD3, MLH1, PALB2, RPE65, COL3A1, DES, ACTC1, APC, WT1, CALM3, NF2, CALM1, CALM2, GLA, BMPR1A, FBN1, OTC.

4. METODOLOGÍA

El ADN genómico obtenido de la muestra obtenida se enriquece en regiones específicas mediante un protocolo basado en hibridación diseñado para cubrir el exoma completo (Twist Bioscience Exome V2.0). Este protocolo permite la detección de variantes genéticas en línea germinal; ofrece alta uniformidad y baja tasa de desvíos ofreciendo datos de calidad. Además, posee una alta cobertura de las principales regiones genéticas codificantes más 20pb flanqueantes a los extremos de los exones según bases de datos internacionales (RefSeq, CCDS, GenCode, Clinvar, ACMG73) y la adición de variantes patogénicas y probablemente patogénicas en regiones no codificantes clínicamente relevantes.

El proceso de secuenciación de siguiente generación se realiza utilizando Tecnología PACBIO de lecturas cortas (*Onso short-read sequencing system*) por medio de tecnología de secuenciación por unión (SBB™). A menos que se indique lo contrario, todas las regiones objetivo se secuencian con una profundidad ≥100x. El procedimiento de Mapeo, Alineamiento y llamado de variantes se realiza utilizando la secuencia de referencia (GRCh38) y los cambios de secuencia se identifican e interpretan según predictores bioinformáticos (Bitgenia) basados en las buenas prácticas establecidas por el Broad Institute (Eli and Edythe L. Broad Institute of Harvard and MIT). El análisis del archivo de variantes (VCF) se realiza mediante el software B-platform.

Teniendo en cuenta el diagnóstico clínico y el modelo de enfermedad propuesto se priorizan variantes utilizando la información disponible en bases de datos (como GnomAD, ClinVar, OMIM, PubMed, LOVD, dbSNP, NCBI Genome, RefSeqGene, entre otros). Las variantes priorizadas se clasifican según la guía internacional de ACMG/AMP[5].

5. LIMITACIONES

Existe la posibilidad de la presencia de variante/s que esté/n causando la afección del paciente que no se detecte/n con este ensayo debido a las limitaciones metodológicas en el presente estudio en el cual no se puede analizar el 100% de la secuencia de los genes (regiones intrónicas altamente repetitivas).

El informe se genera en base a la información disponible al día de la fecha en bases de datos biológicas y publicaciones científicas. Por lo tanto, podría existir una variante actualmente no relacionada al fenotipo del paciente que, en el futuro, podría reclasificarse como causante de la enfermedad. Las variantes halladas en este estudio no han sido confirmadas mediante un método alternativo.

Esta metodología no está optimizada para detectar rearreglos estructurales balanceados (inversiones, translocaciones, etc.), ni para detectar expansiones de trinucleótidos o variantes que se encuentren en regiones de alta complejidad de secuencia (STRs, duplicaciones segmentarias, etc.). Además, este estudio puede no ser concluyente si las variantes se presentan en mosaicos germinales o se encuentran restringidas a un tejido específico.

Tabla 1	
**Library Prep Kit**	Twist Library Preparation EF Kit 2.0<br>Twist Target Enrichment Standard Hybridization v2 Protocol
**Referencia**	GRCh38
**Variantes**	84181

**Variantes Conocidas**	83841
**Prop. dbSNP**	0.996
**Het/Hom Ratio**	1.28
**Ti/Tv Ratio**	2.38
**Prof. Promedio**	63
**Genes**	16905
**(%)Q40**	98.29

6. REFERENCIAS BIBLIOGRÁFICAS

1. Miller, D. T., Lee, K., Chung, W. K., Gordon, A. S., Herman, G. E., Klein, T. E., ... & ACMG Secondary Findings Working Group documents@ acmg. net. (2021). ACMG SF v3. 0 list for reporting of secondary findings in clinical exome and genome sequencing: a policy statement of the American College of Medical Genetics and Genomics (ACMG). Genetics in medicine, 23(8), 1381-1390.

2. Richards, S., Aziz, N., Bale, S., Bick, D., Das, S., Gastier-Foster, J., Grody, W.W., Hegde, M., Lyon, E., Spector, E., et al.; ACMG Laboratory Quality Assurance Committee (2015). Standards and guidelines for the interpretation of sequence variants: a joint consensus recommendation of the American College of Medical Genetics and Genomics and the Association for Molecular Pathology. Genet. Med. 17, 405–424.

3. National Center for Biotechnology Information. ClinVar; [VCV000265202.44], https://www.ncbi.nlm.nih.gov/clinvar/variation/VCV000265202.44 (accessed Sept. 12, 2025).

4. Mütze U, Reischl-Hajiabadi A, Kölker S. Classic Isovaleric Acidemia. 2024 Mar 14. In: Adam MP, Feldman J, Mirzaa GM, et al., editors. GeneReviews® [Internet]. Seattle (WA): University of Washington, Seattle; 1993-2025. Available from: https://www.ncbi.nlm.nih.gov/books/NBK601614/

5. Entry - #617271 - NEPHRONOPHTHISIS 20; NPHP20 - OMIM - (OMIM.ORG) (2017) Omim.org. Available at: https://omim.org/entry/617271 (Accessed: 12 September 2025).

6. National Center for Biotechnology Information. ClinVar; [VCV001321432.7], https://www.ncbi.nlm.nih.gov/clinvar/variation/VCV001321432.7 (accessed Sept. 12, 2025).
